# Nitrogen-Doped Hierarchical Porous Activated Carbon Derived from Paddy for High-Performance Supercapacitors

**DOI:** 10.3390/ma14020318

**Published:** 2021-01-09

**Authors:** Yudan Yuan, Yi Sun, Zhichen Feng, Xingjian Li, Ruowei Yi, Wei Sun, Cezhou Zhao, Li Yang

**Affiliations:** 1 School of Electronic and Information Engineering, Xi’an Jiaotong University, Xi’an 710049, China; yyd899@stu.xjtu.edu.cn; 2 Department of Electrical and Electronic Engineering, Xi’an Jiaotong-Liverpool University, Suzhou 215123, China; yi.sun@xjtlu.edu.cn (Y.S.); zhichen.feng19@student.xjtlu.edu.cn (Z.F.); xingjian.li14@student.xjtlu.edu.cn (X.L.); 3 Department of Electrical Engineering and Electronics, University of Liverpool, Liverpool L69 3GJ, UK; 4 Department of Chemistry, Xi’an Jiaotong-Liverpool University, Suzhou 215123, China; ruowei.yi@xjtlu.edu.cn; 5 Department of Chemistry, University of Liverpool, Liverpool L69 7ZD, UK; 6 GMCC Electronic Technology Wuxi Co. Ltd., Wuxi 214000, China; wei.sun@hypcap.com

**Keywords:** hierarchical porous carbon, paddy, KHCO_3_, dicyandiamide, supercapacitors

## Abstract

A facile and environmentally friendly fabrication is proposed to prepare nitrogen-doped hierarchical porous activated carbon via normal-pressure popping, one-pot activation and nitrogen-doping process. The method adopts paddy as carbon precursor, KHCO_3_ and dicyandiamide as the safe activating agent and nitrogen dopant. The as-prepared activated carbon presents a large specific surface area of 3025 m^2^·g^−1^ resulting from the synergistic effect of KHCO_3_ and dicyandiamide. As an electrode material, it shows a maximum specific capacitance of 417 F·g^−1^ at a current density of 1 A·g^−1^ and very good rate performance. Furthermore, the assembled symmetric supercapacitor presents a large specific capacitance of 314.6 F·g^−1^ and a high energy density of 15.7 Wh·Kg^−1^ at 1 A·g^−1^, maintaining 14.4 Wh·Kg^−1^ even at 20 A·g^−1^ with the energy density retention of 91.7%. This research demonstrates that nitrogen-doped hierarchical porous activated carbon derived from paddy has a significant potential for developing a high-performance renewable supercapacitor and provides a new route for economical and large-scale production in supercapacitor application.

## 1. Introduction

As a new kind of energy storage device, supercapacitors have been a key technology and research focus in the energy industry. It is generally recognized that the electrode materials are critical for developing high-performance supercapacitors. Among various electrode materials, activated carbon is the most common electrode material used for supercapacitors on account of its large specific surface area, stable chemical properties and low cost, while the low energy density limits its practical applications [1,2]. Therefore, increasing the specific surface area, as well as energy density, is a significant step towards meeting growing energy demand.

Recently, plenty of research work has been devoted to enhancing the performance of activated carbon electrode materials. Activated carbon synthesized with various conducting polymers [3,4] and transition metal oxides [5,6] enhances the specific capacitance by reducing contact resistance and inducing extra pseudo-capacitance. Hierarchical porous carbon materials prepared by template-assisted methods have optimized carbon structures, because the macrospores and mesopores provide channels to facilitate ion transport, while micropores are more favored to form the electrical double layers [7,8]. Chemical activation agents, such as H_3_PO_4_, KOH or ZnCl_2_, are also employed to prepare porous carbons with ordered pore structures [9,10,11,12]. Despite their excellent capacitance performance, they are usually confronted with complex processes, like dangerous chemicals, harmful by-products, and expensive cost. Besides, the introduction of heteroatoms, especially nitrogen into carbon materials, has been considered as another efficient technique to improve the electrochemical performance of carbon-based supercapacitors. It is reported that the incorporation of nitrogen could create more active sites, adjust the electron donor properties of the carbon materials, and improve the interface characteristics between the electrode material and electrolytes [13,14,15,16]. Therefore, exploiting an economical and green technique to prepare porous carbon materials doped by nitrogen has attracted significant interest for preparing supercapacitor electrodes.

Using biomass as raw materials, the popping process has been adopted extensively to prepare three-dimensional carbon material using the Chinese popcorn machine. When the temperature and pressure are high enough, the lid of the container is opened, so the differential pressure between the inside and outside of the rice increases rapidly, and the high-pressure steam in the rice expands rapidly, leading the rice to explode into puffed rice. Lai et al. reported the fabrication of dual-heteroatom-doped carbon nanosheets derived from polished rice and wheat via Chinese popcorn machine for supercapacitor and lithium-ion battery applications [17]. Popcorn-derived hierarchical honeycomb-like porous carbon fabricated by applying a steam-explosion method and KOH activation exhibited sizeable specific surface area, high porosity, and was nitrogen-incorporated [18].

**Figure 1:**
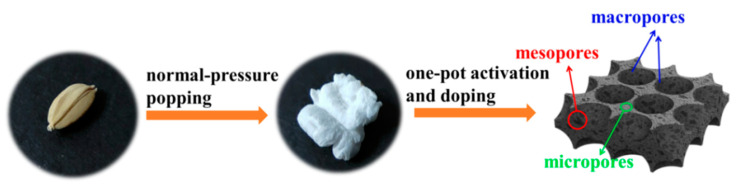
A schematic illustration of the preparation of nitrogen-doped hierarchical porous activated carbon.

Inspired by the Chinese popcorn machine, a facile and green fabrication of nitrogen-doped hierarchical porous activated carbon derived from paddy via normal-pressure popping, one-pot activation and nitrogen-doping process, has been demonstrated (Figure 1). Compared with previous research, two crucial points are proposed: (1) the popping principle of this experiment is similar to that of the traditional popcorn machine. In the process of heating the paddy with a dense husk, the water inside the rice forms vapor, which expands and breaks through the husk to form puffed rice. Because there is no need for a high-pressure vessel, the method is safer and more convenient than the traditional popcorn machine. (2) The synergistic effect of KHCO_3_ and dicyandiamide constructs a hierarchical porous structure and also introduces nitrogen atoms to the carbon materials successfully. The obtained material presents a large specific surface area of 3025 m^2^·g^−1^, which brings a maximum specific capacitance of 417 F·g^−1^ (at 1 A·g^−1^), and exhibits an excellent rate performance too. In addition, the assembled supercapacitor presents a specific capacitance of 314.6 F·g^−1^, while it has a capacitance retention of 93.3% after 10,000 cycles, which reveals a superb long-term cycle stability, and a remarkable energy density of 15.7 Wh·Kg^−1^ with 91.7% retention from 1~20 A·g^−1^. These performances are superior to most previous reports on biomass-derived carbon materials.

## 2. Materials and Methods

### 2.1. Material Synthesis

An appropriate number of paddies were added to a heated pot and stirred continuously for a few minutes. After the paddy exploded, the rice husk was removed with a sieve. Subsequently, puffed rice, KHCO_3_ and dicyandiamide were added to appropriate deionized water according to the mass ratio of 1:3:1, respectively, and then fully stirred and baked at 75 °C for 48 h. Afterwards, the dried mixture was put into a tubular furnace under Ar atmosphere at 800 °C for 2 h with the heating rate of 5 °C/min, and then naturally cooled down to room temperature. Finally, the activated product was washed by using diluted hydrochloric acid to get rid of the residual potassium derivatives and other impurities in the product. Then it was repeatedly filtered with deionized water to neutral. The obtained sample was labeled as PRK3N1. In order to analyze the interaction between KHCO_3_ and dicyandiamide, pure puffed rice (labeled as PR), puffed rice mixed with KHCO_3_ (labeled as PRK3), and puffed rice mixed with dicyandiamide (labeled as PRN1) were prepared under the same processes and conditions, respectively. A more detailed recipe of all samples is shown in Table 1.

### 2.2. Material Characterizations

Scanning electron microscopy (SEM) images of the samples were recorded on a Hitachi SU8010 SEM system (Hitachi, Tokyo, Japan). Transmission electron microscopy (TEM) images were performed on FEI Tecnai G2 F20 S-twin TEM system (FEI, Hillsboto, OR, USA). Raman spectrums were acquired by using a Renishaw inVia Raman microscope (Renishaw, Gloucestershire, UK). A Bruker D8 ADVANCE diffractometer (Bruker, Karlsruhe, Germany) with Cu Kα radiation was used to analyze X-ray diffraction (XRD) patterns. X-ray photoelectron spectroscopy (XPS) analysis was examined by an ESCALAB 250Xi system (Thermo Fisher Scientific, Waltham, MA, USA). The specific surface areas and pore size distribution of the samples were measured by the Brunauere–Emmette–Teller (BET) method on a MicroActive ASAP instrument (Micromeritics, Norcross, GA, USA).

### 2.3. Electrochemical Testing

The electrochemical properties of the obtained materials were evaluated in two-electrode and three-electrode systems, respectively, using 6 M KOH aqueous solution as the electrolyte. To fabricate the working electrode, the active materials, polyvinylidene fluoride and carbon black were mixed according to the mass ratio 8:1:1 in moderate N-methyl-2-pyrrolidone solvent. After stirring for 6 h, the slurry was coated on a nickel foam and vacuum baked at 80 °C for 24 h. The mass loading of the active material for a single electrode was about 3 mg·cm^−2^. An Hg/HgO electrode and a platinum foil were used as the reference electrode and counter electrode in the three-electrode system. In the two-electrode system, two PRK3N1 electrodes were employed to assemble the symmetric supercapacitor with a separator. A Metrohm Autolab PGSTAT302N electrochemical workstation (Metrohm, Herisau, Switzerland) was applied to carry out galvanostatic charge/discharge (GCD), Cyclic voltammetry (CV), and electrochemical impedance spectroscopy (EIS) tests.

The specific capacitance (C_g_) was calculated by the following Equation (1) according to the discharge curve:

C_g_ = k I ∆t/(m ∆V) (1)

where k = 1 in a three-electrode system and k = 2 in a two-electrode system, I (A) is the discharge current, ∆t (s) the discharge time, m (g) is the mass of active materials in each electrode, and ∆V (V) is the discharge voltage range removal of the IR drop [19,20].

The power density (P) and energy density (E) for the two-electrode system were calculated by the following Equations (2) and (3):

E = C (∆V) ^2^/8 (2)

P = E/∆t(3)

where C (F·g^−1^) is the specific capacitance in the two-electrode system calculated from the Equation (1), ∆t (s) is the discharge time, and ∆V (V) is the discharge voltage range excluding the IR drop [21,22].

## 3. Results and Discussions

**Figure 2:**
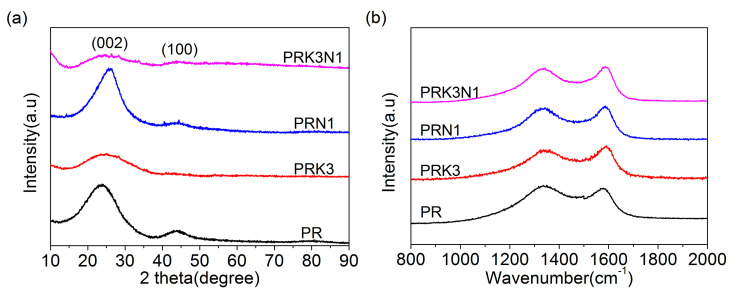
(**a**) XRD patterns, and (**b**) Raman spectrums of PR, PRK3, PRN1 and PRK3N1.

The XRD patterns of PR, PRK3, PRN1, PRK3N1 are shown in Figure 2a. All samples exhibit two wide peaks near 25°and 43°, which could be matched with (002) and (100) planes of the graphitic structure [23]. By contrast, the peak intensities of PRK3 and PRK3N1 are much weaker than those of PR and PRN1, suggesting that the graphitization degrees of PRK3 and PRK3N1 are much lower due to the increase of the disorder after KHCO_3_ activation. The structural characteristics and graphitization degrees could be further confirmed by Raman spectroscopy analysis. Figure 2b shows the Raman spectrum of all samples. The D band at 1350 cm^−1^ presents the disordered and defective structures of the carbon lattice, while G band at 1580 cm^−1^ is related to the in-plane tensile vibration of sp^2^-hybridized carbon atoms of graphite [24,25]. Generally, the relative intensity ratio of D-band to G-band (I_D_/I_G_) reveals the degree of graphitization and defect of carbon materials. The I_D_/I_G_ values of PR, PRK3, PRN1 and PRK3N1 are 2.57, 2.70, 2.59, and 2.71, respectively. The largest I_D_/I_G_ value of PRK3N1 indicates the highest degree of disorder, which can mainly be ascribed to the heteroatom doping and hierarchical porous structure, which may introduce more electrochemical active sites and enhance the capacitance performance [14].

**Figure 3:**
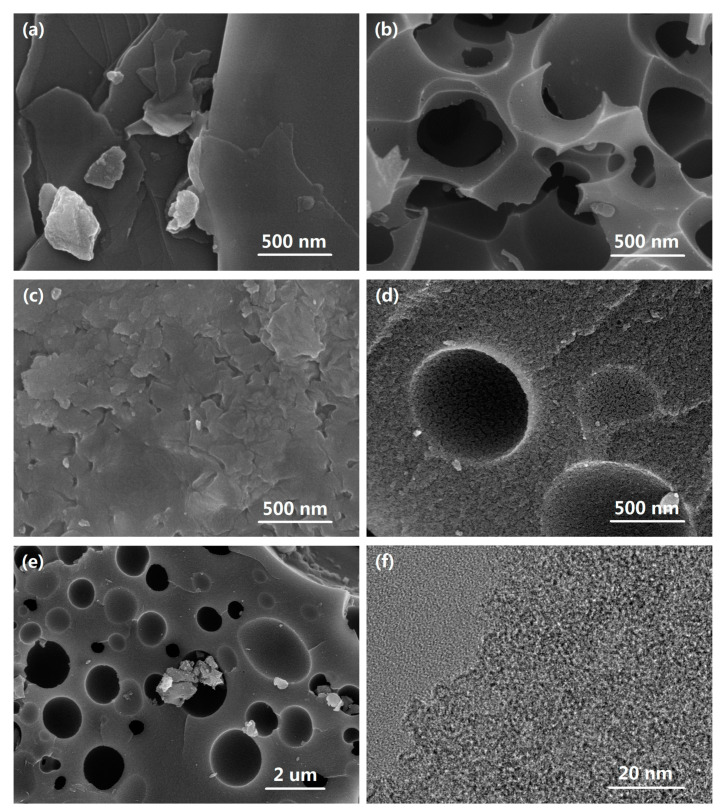
SEM images of (**a**) PR, (**b**) PRK3, (**c**) PRN1, and (**d**,**e**) PRK3N1 with different magnifications, and (**f**) TEM image of PRK3N1.

The micro-structure and morphology of all the samples are displayed in Figure 3. According to Figure 3a,c, PR and PRN1 present stacked-layer morphology with a smooth surface, demonstrating that there are barely any pores on the surface. The SEM image of PRK3 shown in Figure 3b, exhibits porous frameworks with abundant macropores owing to the activation of KHCO_3_. By comparison, PRK3N1 (Figure 3d,e) displays more regular macropores and rough morphology, which implies that more micropores and mesopores are created after the synergistic effect of KHCO_3_ and dicyandiamide [26,27]. In addition, highly disordered structure of PRK3N1 is presented in TEM image (Figure 3f), revealing its amorphous structure, which is in accord with the characteristic peaks in XRD pattern.

**Figure 4:**
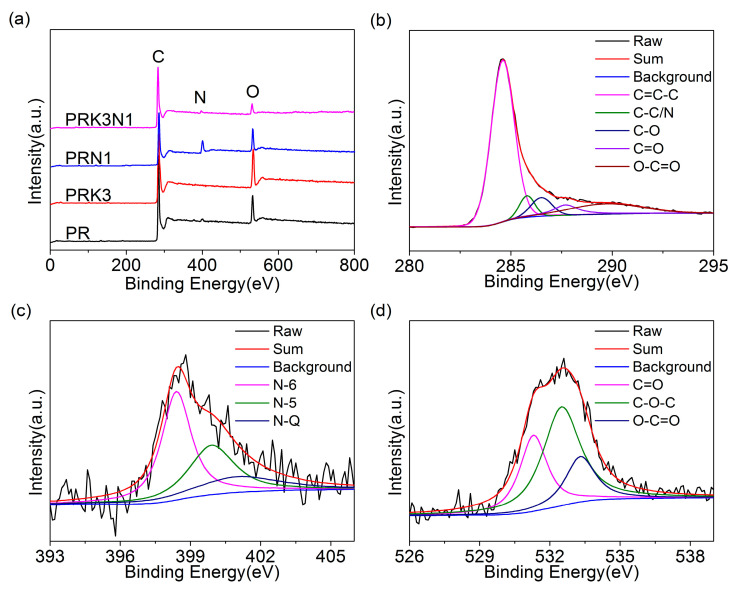
(**a**) Full XPS spectra of PR, PRK3, PRN1 and PRK3N1, and high-resolution (**b**) C 1s, (**c**) N 1s and (**d**) O 1s of PRK3N1.

An in-depth study was applied to analyze the atomic compositions and surface chemical properties of all samples, the fully scanned XPS spectra are shown in Figure 4a, displaying three peaks at 284, 399 and 533 eV corresponded to C 1*s*, N 1*s* and O 1*s*, respectively, further confirming the presence of abundant heteroatom in all samples. The contents of carbon, nitrogen and oxygen are seen in Table 2. The atomic percentage of nitrogen in PRN1 (13.38%) and PRK3N1 (4.26%) is higher than that of PR (3.34%) and PRK3 (not detected), respectively, which indicates that dicyandiamide has successfully introduced nitrogen atoms to the carbon materials. To analyze the electronic states of PRK3N1, the high-resolution C 1*s*, N 1*s* and O 1*s* spectra are presented in Figure 4b–d. The C 1*s* spectrum could be approximately fitted with five peaks, which could be matched with C=C-C bond (284.6 eV), C-C/C-N bond (285.8 eV), C-O bond (286.5 eV), C=O bond (287.7eV), and O-C=O band (289.7 eV), respectively [28,29]. The high-resolution N 1*s* spectrum could be deconvoluted into three peaks centered at 398.4 eV, 399.9 eV and 401.0 eV, related to the pyridinic-N (N-6), pyrrolic-N (N-5) and quaternary-N (N-Q), respectively [17,30]. It is noteworthy that pyridinic-N and pyrrolic-N have good electron-donor properties to provide pseudo-capacitance, while the quaternary-N could favor the transfer of electrolyte ions and improve the conductivity of carbon materials [15,31]. Furthermore, the O 1*s* spectrum is well-matched with three peaks, which are attributed to C=O (531.3 eV), C-O-C (532.5 eV) and O-C=O (533.3 eV), respectively [22]. Besides additional pseudo-capacitance, these hydrophilic functional groups could promote the wettability of the interface between the carbon materials and the electrolyte, which is beneficial to forming electrochemical double layers.

**Figure 5:**
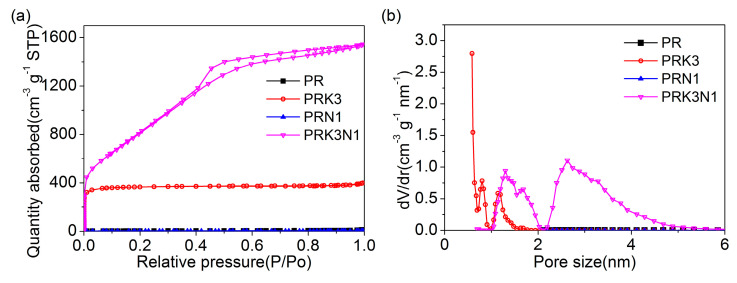
(**a**) Nitrogen absorption–desorption isotherms, (**b**) the pore size distribution curves of PR, PRK3, PRN1 and PRK3N1.

The nitrogen adsorption–desorption measurements were performed to estimate the porous structure of all samples. According to Figure 5a, the isotherms of PR and PRN1 are close to the horizontal axis, implying there are few pores in these two samples, which is in accordance with the results of SEM (Figure 3a,c). After KHCO_3_ activation, PRK3 possesses typical type I adsorption–desorption isotherms with a steep increase at very low relative pressure, indicating the existence of abundant micropores [11,32]. The isotherm of PRK3N1 has a higher sharp rise, followed by an obvious increase and hysteresis loop at the high relative pressures, suggesting that PRK3N1 possesses more micropores than PRK3 and also a large amount of mesopores [33,34]. More details of the pore structure of all samples are listed in Table 3. It can be observed that PR and PRN1 have extremely low surface area/pore volume (11 m^2^·g^−1^/0.018 cm^3^·g^−1^, 6 m^2^·g^−1^/0.003 cm^3^·g^−1^, respectively), while PRK3 synthesized by KHCO_3_ activation has a higher surface area (1436 m^2^ g^−1^) and larger pore volume (0.614 cm^3^·g^−1^), which is attributed to the intense activation properties of KHCO_3_. As previously reported, after KHCO_3_ is completely decomposed at about 200 °C, the formation of CO_2_ and H_2_O (Equation (4)) positively contributes to creating the macropores [23]. When the temperature reaches 800 °C, the carbon framework is etched by the redox reactions described in Equations (5)–(8), leading to abundant micropores after the removal of metallic K and other K compounds by washing [35]. However, PRK3N1 presents the highest surface area (3025 m^2^·g^−1^) and largest pore volume (2.381 cm^3^·g^−1^), suggesting that the activation ability is obviously strengthened as the simultaneous presence of KHCO_3_ and dicyandiamide at high temperature, although the reaction mechanism is still unclear and needs to be further verified.

2KHCO_3_ → K_2_CO_3_ + CO_2_ + H_2_O (4)

K_2_CO_3_ + 2C → 2K + 3CO (5)

K_2_CO_3_ → K_2_O + CO_2_ (6)

CO_2_ + C → 2CO (7)

K_2_O + C → 2K + CO (8)

The pore size distribution (PSD) of all samples measured by the NLDFT model is shown in Figure 5b. The pore size of PRK3 is concentrated in the scope of 0.5–2.0 nm, whereas PRK3N1 possesses a high proportion of mesopores with a pore size from 2.0 to 5.0 nm, which is consistent with the previous results. Thus, the PRK3N1 presents a hierarchical porous structure composed of micro-, meso-, and macropores, which could improve ion transport in the electrolyte.

From the above discussion, the conclusion can be drawn that the synergistic effect of KHCO_3_ and dicyandiamide has a significant effect on forming a hierarchical porous architecture and doping nitrogen atoms, which is propitious to improving the electrochemical performance of the as-prepared materials.

**Figure 6:**
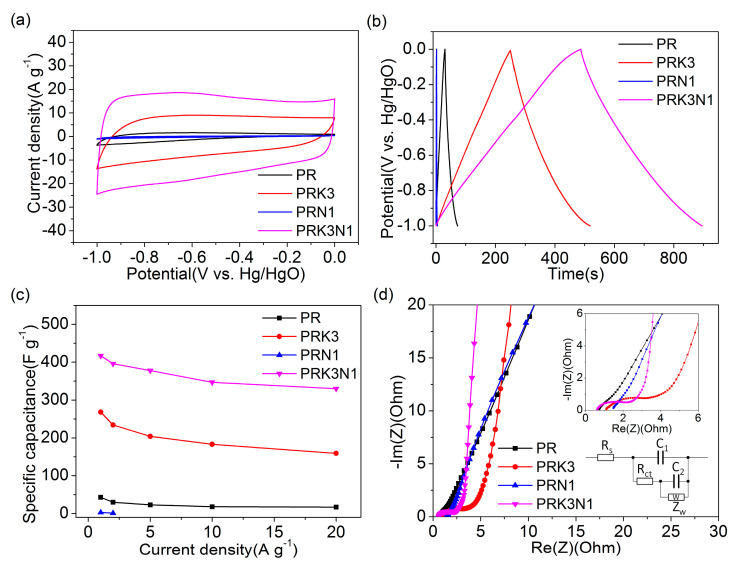
The electrochemical properties of all samples measured in a three-electrode system: (**a**) the CV curves of PR, PRK3, PRN1 and PRK3N1 at 50 mV·s^−1^; (**b**) the GCD curves of PR, PRK3, PRN1 and PRK3N1 at 1 A·g^−1^; (**c**) the specific capacitances of PR, PRK3, PRN1 and PRK3N1 at different current densities; (**d**) the Nyquist plots of all samples. The inset window shows the equivalent circuit and an enlarged view at high frequency.

The CV curves of PR, PRK3, PRN1 and PRK3N1 at the scan rate of 50 mV·s^−1^ are displayed in Figure 6a. All CV curves show rectangular-like shapes with a typical feature of double-layer supercapacitor behavior. The distortion of the CV curves can be ascribed to the redox reactions of nitrogen and oxygen-containing functional groups, indicating the existence of pseudo-capacitance [36,37]. Compared with other samples, PRK3N1 presents the largest integrated area and the most rectangular-like shape, which is indicative of the best capacitor behavior. The most probable reason is that PRK3N1 has a large number of meso- and macropores, which provide routes for the fast transport of electrolyte ions, leading to rapid current response during voltage conversion.

The GCD curves of all samples measured at a current density of 1 A·g^−1^ are illustrated in Figure 6b. It is clearly observed that PRK3N1 has the longest charge–discharge time. The roughly triangular shapes further confirm the contribution of pseudo-capacitance [38], which is consistent with the CV results. To further estimate the rate performance the calculated specific capacitances from the GCD profiles of all samples are summarized in Figure 6c (The specific capacitance of PRN1 is too small to be measured at high current density). PRK3N1 exhibits the highest specific capacitance of 417 F·g^−1^ at 1 A·g^−1^, with the capacitance retention of 79% even at 20 A·g^−1^ (330 F·g^−1^). The high specific capacitance and outstanding rate capability of PRK3N1 may be attributed to the hierarchical porous structure and heteroatomic doping resulting from the coordinative impact of KHCO_3_ and dicyandiamide, which would further induce extra pseudo-capacitance, shorten the ion diffusion pathway and create more active sites in favor of rapid charge transfer [39].

The Nyquist plots of all samples tested in the range of 10 mHz to 100 kHz with 5 mV voltage amplitude are shown in Figure 6d. The almost vertical curves of PRK3 and PRK3N1 indicate an ideal capacitive performance and an excellent ion diffusion and migration behavior in the low-frequency region [1,40]. To further understand the electrochemical impedance and charge–transport characteristics of PRK3 and PRK3N1, a circuit simulation was carried out with the equivalent circuit model [41] shown in inset of Figure 6d. In the model, R_s_ represents the serial resistance related to the electrolyte, R_ct_ represents the charge transfer resistance, Z_w_ represents the Warburg impedance arising from diffusion limitation, while C_1_ and C_2_ represent the pseudo-capacitance and electrical double-layer capacitance, respectively. The simulation results of PRK3 and PRK3N1 are shown in Table S1 and Figure S1. It can be seen that the serial resistance R_s_ values are 1.08 Ω and 0.50 Ω for PRK3 and PRK3N1, respectively. Meanwhile, the charge transfer resistance R_ct_ of PRK3N1(2.12 Ω) is smaller than that of PRK3 (2.5 Ω), which implies the good conductivity and rapid charge transfer of PRK3N1 is due to efficient transport channels brought by abundant mesopores and macropores [36].

**Figure 7:**
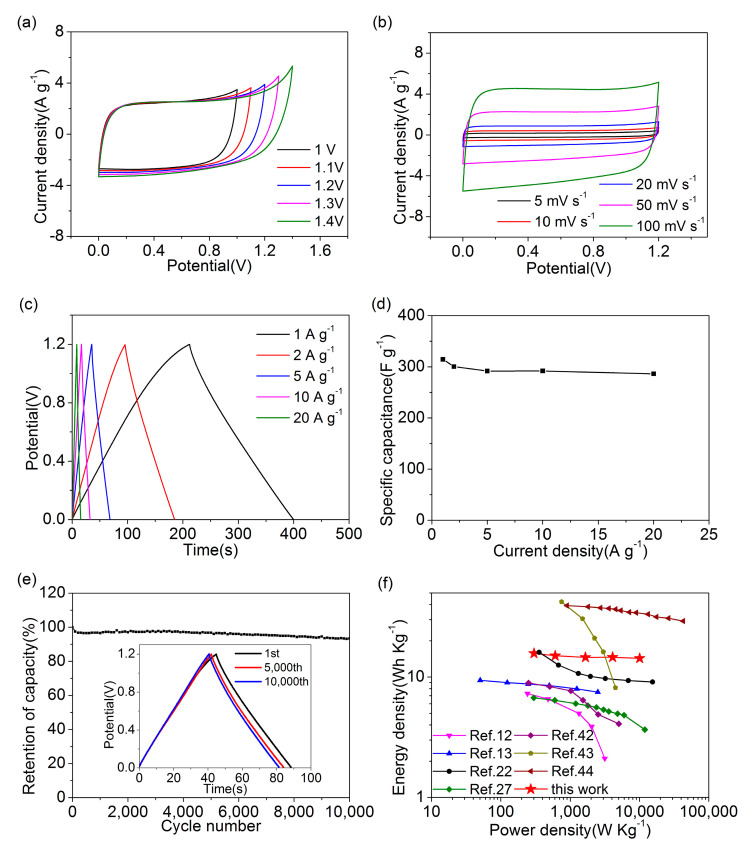
The electrochemical characteristics of symmetric supercapacitor of PRK3N1: (**a**) CV curves at 50 mV·s^−1^ with different potential windows; (**b**) the CV curves at various scan rates from 5 to 100 mV·s^−1^; (**c**) the GCD curves from 1 to 20 A·g^−1^; (**d**) the specific capacitances of symmetric supercapacitor from 1 to 20 A·g^−1^; (**e**) cycle stability at 4 A·g^−1^, the GCD curves of the 1st cycle, 5000th cycle and 10,000th cycle are shown in the inset; (**f**) the Ragone plots of PRK3N1 and other reported symmetric supercapacitors.

In order to evaluate the practical electrochemical performance of PRK3N1, a symmetric supercapacitor was assembled by applying two PRK3N1 electrodes and measured in 6 M KOH electrolyte. The CV curves of PRK3N1 obtained from different potential ranges are shown in Figure 7a. It can be seen that no obvious distortion is displayed until 1.2 V, demonstrating that 0-1.2 V is the optimum voltage range of the assembled supercapacitor. The CV curves from 5 to 100 mV·s^−1^, and the GCD curves from 1 to 20 A·g^−1^, are shown in Figure 7b,c, respectively, indicating a representative electrochemical supercapacitor behavior. According to the GCD profiles, the specific capacitance of PRK3N1 is calculated as 314.6 F·g^−1^ at the current density of 1 A·g^−1^, and 286.2 F·g^−1^ at 20 A·g^−1^ (Figure 7d), demonstrating an excellent specific capacitance and rate performance, which again confirms the effective synergistic combination of KHCO_3_ and dicyandiamide. Furthermore, in order to investigate the long-term cycling stability, a measurement of the symmetric supercapacitor was applied by the GCD test at a constant current density of 4 A·g^−1^. Figure 7e demonstrates that the device exhibits outstanding cycle stability with 93.3% retention after 10,000 cycles, which suggests a great potential in energy storage applications.

The Ragone plot, which depicts the relationship between power density and energy density, is shown in Figure 7f. The symmetric supercapacitor based on PRK3N1 exhibits a maximum energy density of 15.7 Wh·Kg^−1^ at 1 A·g^−1^. Furthermore, the energy density is retained at 14.4 Wh·Kg^−1^ with 91.7% retention as the current density increases to 20 A·g^−1^. Compared with the previously reported symmetric supercapacitors derived from biomass measured in 6 M KOH electrolyte [12,13,22,27] and PVA-KOH gel electrolyte [42], the obtained energy density of the PRK3N1 supercapacitor presents a better and more stable property than others. Meanwhile, composite MnO_2_/porous carbon materials [43] showed a higher energy density than PRK3N1, which resulted from the larger potential window of 1.5 V in 1 M Na_2_SO_4_ electrolyte. However, the energy density decayed rapidly due to the poor rate performance. Furthermore, cross-linked holey graphene/porous carbon [44] exhibited the best energy density in Figure 7f, which may be attributed to the conductive network provided by the special structure of graphene/casein and more heteroatom doping. Above all, nitrogen-doped hierarchical porous activated carbon derived from paddy has a broad prospect for developing high-performance supercapacitors.

## 4. Conclusions

In summary, nitrogen-doped hierarchical porous activated carbon derived from paddy was synthesized via normal-pressure popping, one-pot activation and nitrogen-doped process. Due to the synergistic effect of KHCO_3_ and dicyandiamide, the as-obtained activated carbon exhibits a large surface area, heteroatom doping and hierarchical porous construction. The electrochemical performance of PRK3N1 presents a high specific capacitance of 417 F·g^−1^ (at 1 A·g^−1^) and possesses an outstanding rate performance. Furthermore, the assembled symmetric supercapacitor presents a high specific capacitance of 314.6 F·g^−1^ and a superb capacitance retention of 93.3% after 10,000 cycles, as well as remarkable energy density with 91.7% retention from 1 to 20 A·g^−1^. The prominent capacitor behavior illustrates that nitrogen-doped hierarchical porous activated carbon derived from paddy has a brilliant foreground in high-performance renewable energy storage devices.

## Figures and Tables

**Table 1:** A detailed recipe of all samples.

Samples	Puffed Rice(g)	KHCO_3_(g)	Dicyandiamide(g)
PR	5	--	--
PRK3	5	15	--
PRN1	5	--	5
PRK3N1	5	15	5

**Table 2:** The chemical compositions of all samples by XPS analysis.

Element	PR	PRK3	PRN1	PRK3N1
C (Atom%)	83.16	82.41	73.03	88.62
N (Atom%)	3.34	--	13.38	4.26
O (Atom%)	12.47	17.59	12.67	7.12
P (Atom%)	1.03	--	0.92	--

**Table 3:** The pore structural parameters of all samples.

Samples	S_BET_^1^ (m^2^·g^−1^)	S_micro_^2^ (m^2^·g^−1^)	V_total_^3^ (cm^3^·g^−1^)	V_micro_^4^ (cm^3^·g^−1^)
PR	11	5	0.018	0.002
PRK3	1436	1422	0.614	0.566
PRN1	6	5	0.003	0.002
PRK3N1	3025	1855	2.381	1.260

^1^ The specific surface area computed by Brunauere–Emmette–Teller (BET) equation. ^2^ The specific surface area of micropores acquired by t-plot method. ^3^ The total pore volume measured when the relative pressure is 0.99. ^4^ The pore volume of micropores obtained from the t-plot method.

## Data Availability

Data sharing not applicable.

## Supplementary Materials

The following are available online at https://www.mdpi.com/1996-1944/14/2/318/s1, Figure S1: The fitted curves of PRK3 and PRK3N1, Table S1: the simulation values of each element of PRK3 and PRK3N1 fitted in the equivalent circuit.

## References

[1] Wang C., Wu D., Wang H., Gao Z., Xu F., Jiang K. (2018). A·green and scalable route to yield porous carbon sheets from biomass for supercapacitors with high capacity. J. Mater. Chem. A.

[2] Qie L., Chen W., Xu H., Xiong X., Jiang Y., Zou F., Hu X., Xin Y., Zhang Z., Huang Y. (2013). Synthesis of functionalized 3D hierarchical porous carbon for high-performance supercapacitors. Energy Environ. Sci..

[3] Lee J.W., Lee H.I., Park S.-J. (2018). Facile synthesis of petroleum-based activated carbons/tubular polypyrrole composites with enhanced electrochemical performance as supercapacitor electrode materials. Electrochim. Acta.

[4] Khan S., Majid A., Raza R. (2020). Synthesis of PEDOT: PPy/AC composite as an electrode for supercapacitor. J. Mater. Sci.-Mater. Electron..

[5] Kim S.C., Park Y.K., Kim B.J., An K.H., Lee W.J., Lee H., Jung S.C. (2019). Facile synthesis of chromium oxide on activated carbon electrodes for electrochemical capacitor application. J. Nanosci. Nanotechnol..

[6] Li J., Wang Y.W., Xu W.N., Wang Y., Zhang B., Luo S., Zhou X.Y., Zhang C.L., Gu X., Hu C.G. (2019). Porous Fe_2_O_3_ nanospheres anchored on activated carbon cloth for high-performance symmetric supercapacitors. Nano Energy.

[7] Zhao Q.L., Wang X.Y., Liu J., Wang H., Zhang Y.W., Gao J., Lu Q., Zhou H.Y. (2015). Design and synthesis of three-dimensional hierarchical ordered porous carbons for supercapacitors. Electrochim. Acta.

[8] Yao L., Yang G.Z., Han P., Tang Z.H., Yang J.H. (2016). Three-dimensional beehive-like hierarchical porous polyacrylonitrile-based carbons as a high performance supercapacitor electrodes. J. Power Sources.

[9] Sun K.J., Zhang Z.G., Peng H., Zhao G.H., Ma G.F., Lei Z.Q. (2018). Hybrid symmetric supercapacitor assembled by renewable corn silks based porous carbon and redox-active electrolytes. Mater. Chem. Phys..

[10] Inal I.I.G., Holmes S.M., Banford A., Aktas Z. (2015). The performance of supercapacitor electrodes developed from chemically activated carbon produced from waste tea. Appl. Surf. Sci..

[11] Yuan Y.D., Yi R.W., Sun Y., Zeng J.Q., Li J.Q., Hu J.H., Zhao Y.C., Sun W., Zhao C., Yang L. (2018). Porous activated carbons derived from pleurotus eryngii for supercapacitor applications. J. Nanomater..

[12] Zhao Y.Q., Lu M., Tao P.Y., Zhang Y.J., Gong X.T., Yang Z., Zhang G.Q., Li H.L. (2016). Hierarchically porous and heteroatom doped carbon derived from tobacco rods for supercapacitors. J. Power Sources.

[13] Li B., Cheng Y., Dong L., Wang Y., Chen J., Huang C., Wei D., Feng Y., Jia D., Zhou Y. (2017). Nitrogen doped and hierarchically porous carbons derived from chitosan hydrogel via rapid microwave carbonization for high-performance supercapacitors. Carbon.

[14] Lin L., Xie H.M., Lei Y., Li R.Z., Liu X.Y., Ou J.K. (2020). Nitrogen source-mediated cocoon silk-derived N, O-doped porous carbons for high performance symmetric supercapacitor. J. Mater. Sci.-Mater. Electron..

[15] Peng H., Ma G., Sun K., Zhang Z., Yang Q., Lei Z. (2016). Nitrogen-doped interconnected carbon nanosheets from pomelo mesocarps for high performance supercapacitors. Electrochim. Acta.

[16] Wang C., Wu D., Wang H., Gao Z., Xu F., Jiang K. (2017). Nitrogen-doped two-dimensional porous carbon sheets derived from clover biomass for high performance supercapacitors. J. Power Sources.

[17] Lai F.L., Zhou G.Y., Li F., He Z.H., Yong D.Y., Bai W., Huang Y.P., Tjiu W.W., Miao Y.E., Pan B.C. (2018). Highly dual-heteroatom-doped ultrathin carbon nanosheets with expanded interlayer distance for efficient energy storage. ACS Sustain. Chem. Eng..

[18] Liang T., Chen C., Li X., Zhang J. (2016). Popcorn-derived porous carbon for energy storage and CO_2_ capture. Langmuir.

[19] Pang L.Y., Zou B., Han X., Cao L.Y., Wang W., Guo Y.P. (2016). One-step synthesis of high-performance porous carbon from corn starch for supercapacitor. Mater. Lett..

[20] Chee W.K., Lim H.N., Zainal Z., Huang N.M., Harrison I., Andou Y. (2016). Flexible graphene-based supercapacitors: A review. J. Phys. Chem. C.

[21] Ouyang T., Cheng K., Yang F., Zhou L.M., Zhu K., Ye K., Wang G.L., Cao D.X. (2017). From biomass with irregular structures to 1D carbon nanobelts: A stripping and cutting strategy to fabricate high performance supercapacitor materials. J. Mater. Chem. A.

[22] Zhou J.Q., Wang M., Li X. (2019). Promising biomass-derived nitrogen-doped porous carbon for high performance supercapacitor. J. Porous Mater..

[23] Deng J., Xiong T.Y., Xu F., Li M.M., Han C.L., Gong Y.T., Wang H.Y., Wang Y. (2015). Inspired by bread leavening: One-pot synthesis of hierarchically porous carbon for supercapacitors. Green Chem..

[24] Qi J., Zhang W., Xu L. (2018). Solvent-free mechanochemical preparation of hierarchically porous carbon for supercapacitor and oxygen reduction reaction. Chemistry.

[25] Hong P., Liu X., Zhang X., Peng S., Wang Z., Yang Y., Zhao R., Wang Y. (2019). Hierarchically porous carbon derived from the activation of waste chestnut shells by potassium bicarbonate (KHCO_3_) for high-performance supercapacitor electrode. Int. J. Energy Res..

[26] Zhan C.Z., Yu X.L., Liang Q.H., Liu W., Wang Y.B., Lv R.T., Huang Z.H., Kang F.Y. (2016). Flour food waste derived activated carbon for high-performance supercapacitors. RSC Adv..

[27] Niu L.Y., Shen C., Yan L.J., Zhang J.H., Lin Y., Gong Y.Y., Li C., Sun C.Q., Xu S.Q. (2019). Waste bones derived nitrogen-doped carbon with high micropore ratio towards supercapacitor applications. J. Colloid Interface Sci..

[28] Sun H.M., He W.H., Zong C.H., Lu L.H. (2013). Template-free synthesis of renewable macroporous carbon via yeast cells for high-performance supercapacitor electrode materials. ACS Appl. Mater. Interfaces.

[29] Peng C., Yan X.B., Wang R.T., Lang J.W., Ou Y.J., Xue Q.J. (2013). Promising activated carbons derived from waste tea-leaves and their application in high performance supercapacitors electrodes. Electrochim. Acta.

[30] Yin Y.Y., Li R.Y., Li Z.J., Liu J.K., Gu Z.G., Wang G.L. (2014). A facile self-template strategy to fabricate three-dimensional nitrogen-doped hierarchical porous carbon/graphene for conductive agent-free supercapacitors with excellent electrochemical performance. Electrochim. Acta.

[31] Xiong S.Q., Fan J.C., Wang Y., Zhu J., Yu J.R., Hu Z.M. (2017). A facile template approach to nitrogen-doped hierarchical porous carbon nanospheres from polydopamine for high-performance supercapacitors. J. Mater. Chem. A.

[32] Long C.L., Chen X., Jiang L.L., Zhi L.J., Fan Z.J. (2015). Porous layer-stacking carbon derived from in-built template in biomass for high volumetric performance supercapacitors. Nano Energy.

[33] Huang J., Wu J.G., Dai F.Y., Li C.M. (2019). 3D honeycomb-like carbon foam synthesized with biomass buckwheat flour for high-performance supercapacitor electrodes. Chem. Commun..

[34] Lian Y.M., Ni M., Zhou L., Chen R.J., Yang W. (2018). Synthesis of biomass-derived carbon induced by cellular respiration in yeast for supercapacitor applications. Chem.-Eur. J..

[35] Wang J.C., Kaskel S. (2012). KOH activation of carbon-based materials for energy storage. J. Mater. Chem..

[36] Feng H.B., Hu H., Dong H.W., Xiao Y., Cai Y.J., Lei B.F., Liu Y.L., Zheng M.T. (2016). Hierarchical structured carbon derived from bagasse wastes: A simple and efficient synthesis route and its improved electrochemical properties for high-performance supercapacitors. J. Power Sources.

[37] Yin H.Y., Lu B.H., Xu Y., Tang D.Y., Mao X.H., Xiao W., Wang D.H., Alshawabkeh A.N. (2014). Harvesting capacitive carbon by carbonization of waste biomass in molten salts. Environ. Sci. Technol..

[38] Andreas H.A., Conway B.E. (2006). Examination of the double-layer capacitance of an high specific-area C-cloth electrode as titrated from acidic to alkaline pHs. Electrochim. Acta.

[39] Yu M., Han Y.Y., Li Y., Li J., Wang L.J. (2019). Improving electrochemical activity of activated carbon derived from popcorn by NiCo_2_S_4_ nanoparticle coating. Appl. Surf. Sci..

[40] Jin Z.Y., Lu A.H., Xu Y.Y., Zhang J.T., Li W.C. (2014). Ionic liquid-assisted synthesis of microporous carbon nanosheets for use in high rate and long cycle life supercapacitors. Adv. Mater..

[41] Wei H., Wang H., Li A., Li H., Cui D., Dong M., Lin J., Fan J., Zhang J., Hou H. (2020). Advanced porous hierarchical activated carbon derived from agricultural wastes toward high performance supercapacitors. J. Alloys Compd..

[42] Bai Q., Li H., Zhang L., Li C., Shen Y., Uyama H. (2020). Flexible Solid-State Supercapacitors Derived from Biomass Konjac/Polyacrylonitrile-Based Nitrogen-Doped Porous Carbon. ACS Appl. Mater. Interfaces.

[43] Vargheese S., Muthu D., Pattappan D., Kavya K.V., Kumar R.T.R., Haldorai Y. (2020). Hierarchical flower-like MnO_2_@nitrogen-doped porous carbon composite for symmetric supercapacitor: Constructing a 9.0 V symmetric supercapacitor cell. Electrochim. Acta.

[44] Jia S., Zang J., Tian P., Zhou S., Cai H., Tian X., Wang Y. (2020). A 3-D covalently crosslinked N-doped porous carbon/holey graphene composite for quasi-solid-state supercapacitors. Microporous Mesoporous Mater..

