# Supplementary material for: Nitrogen-Doped Hierarchical Porous Activated Carbon Derived from Paddy for High-Performance Supercapacitors
Source: Materials (Basel). 2021 Jan 9;14(2):318. doi: 10.3390/ma14020318 (PMC7828036; doi:10.3390/ma14020318)
Supplement: Supplementary file 1 [file materials-14-00318-s001.pdf]

materials

Supplementary Materials

Nitrogen-Doped Hierarchical Porous Activated Carbon Derived from Paddy for High-Performance Supercapacitors

Yudan Yuan [1], Yi Sun [2,3], Zhichen Feng [2,3], Xingjian Li [2,3], Ruowei Yi [4,5], Wei Sun [6], Cezhou Zhao [1,2,5,*] and Li Yang [4,5,*]

1 School of Electronic and Information Engineering, Xi'an Jiaotong University, Xi'an 710049, China; yyd899@stu.xjtu.edu.cn
2 Department of Electrical and Electronic Engineering, Xi'an Jiaotong-Liverpool University, Suzhou 215123, China; yi.sun@xjtlu.edu.cn (Y.S.); zhichen.feng19@student.xjtlu.edu.cn (Z.F.); xingjian.li14@student.xjtlu.edu.cn (X.L.)
3 Department of Electrical Engineering and Electronics, University of Liverpool, Liverpool L69 3GJ, UK
4 Department of Chemistry, Xi'an Jiaotong-Liverpool University, Suzhou 215123, China; ruowei.yi@xjtlu.edu.cn
5 Department of Chemistry, University of Liverpool, Liverpool L69 7ZD, UK
6 GMCC Electronic Technology Wuxi Co. Ltd., Wuxi 214000, China; wei.sun@hypcap.com
* Correspondence: Cezhou.Zhao@xjtlu.edu.cn (C.Z.); Li.Yang@xjtlu.edu.cn (L.Y.)

Citation: Yuan, Y.; Sun, Y.; Feng, Z.; Li, X.; Yi, R.; Sun, W.; Zhao, C.; Yang, L. Nitrogen-Doped Hierarchical Porous Activated Carbon Derived from Paddy for High-Performance Supercapacitors. *Materials* **2021**, *14(2)*, 318. https://doi.org/10.3390/ma14020318

Received: 18 December 2020

Accepted: 6 January 2021

Published: 9 January 2021

Publisher's Note: MDPI stays neutral with regard to jurisdictional claims in published maps and institutional affiliations.

Copyright: © 2021 by the authors. Licensee MDPI, Basel, Switzerland. This article is an open access article distributed under the terms and conditions of the Creative Commons Attribution (CC BY) license (http://creativecommons.org/licenses/by/4.0/).

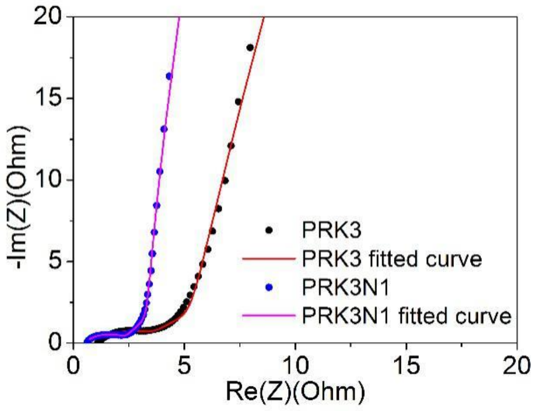

Figure S1. The fitted curves of PRK3 and PRK3N1.

Table S1. The simulation values of each element of PRK3 and PRK3N1 fitted in the equivalent circuit.

Samples	R_s (Ω)	R_{ct} (Ω)	C_1(F)	C_2(F)	Z_w(Ω)
PRK3	1.08	2.50	0.003	0.011	0.73
PRK3N1	0.50	2.12	0.004	0.015	0.19
